# Microorganisms populating the water-related indoor biome

**DOI:** 10.1007/s00253-020-10719-4

**Published:** 2020-06-12

**Authors:** Monika Novak Babič, Cene Gostinčar, Nina Gunde-Cimerman

**Affiliations:** 1 Department of Biology, Biotechnical Faculty, University of Ljubljana, Jamnikarjeva 101, 1000 Ljubljana, Slovenia; 2 Lars Bolund Institute of Regenerative Medicine, BGI-Qingdao, Qingdao, 266555 China

**Keywords:** Household microbiome, water-borne, bacteria, fungi, extremophiles, opportunists

## Abstract

**Abstract:**

Modernisation of our households created novel opportunities for microbial growth and thus changed the array of microorganisms we come in contact with. While many studies have investigated microorganisms in the air and dust, tap water, another major input of microbial propagules, has received far less attention. The quality of drinking water in developed world is strictly regulated to prevent immediate danger to human health. However, fungi, algae, protists and bacteria of less immediate concern are usually not screened for. These organisms can thus use water as a vector of transmission into the households, especially if they are resistant to various water treatment procedures. Good tolerance of unfavourable abiotic conditions is also important for survival once microbes enter the household. Limitation of water availability, high or low temperatures, application of antimicrobial chemicals and other measures are taken to prevent indoor microbial overgrowth. These conditions, together with a large number of novel chemicals in our homes, shape the diversity and abundance of indoor microbiota through constant selection of the most resilient species, resulting in a substantial overlap in diversity of indoor and natural extreme environments. At least in fungi, extremotolerance has been linked to human pathogenicity, explaining why many species found in novel indoor habitats (such as dishwasher) are notable opportunistic pathogens. As a result, microorganisms that often enter our households with water and are then enriched in novel indoor habitats might have a hitherto underestimated impact on the well-being of the increasingly indoor-bound human population.

**Key points:**

- *Domestic environment harbours a large diversity of microorganisms.*
- *Microbiota of water-related indoor habitats mainly originates from tap water.*
- *Bathrooms, kitchens and household appliances select for polyextremotolerant species.*
- *Many household-related microorganisms are human opportunistic pathogens.*

## Introduction

The world we live in is rapidly changing. Clean and safe water is an indispensable resource that is becoming increasingly scarce as the world population is growing, consumption as well as pollution is going up, urbanisation is spreading and mega cities became the new ecological reality. People from developed societies now spend approximately 90% of their lives indoors (Kelley and Gilbert 2013). This increases their exposure to indoor microorganisms, the diversity and abundance of which can differ profoundly from natural environments or even human dwellings of the past. Some of these microorganisms can cause allergies and infectious diseases, in particular in immunocompromised, chronically ill, or elderly individuals as well as infants (de Hoog et al. 2014).

Microorganisms enter buildings with outdoor air, soil, water, living plants and different food products as well as other humans and pets (Weikl et al. 2016; Jayaprakash et al. 2017; Shan et al. 2019; Cooper 2010). Most published overviews of indoor microbiomes focus on air-borne, dust-associated microbes (Shan et al. 2019). Comparatively little has been published on microbes in domestic water sources or in wet or humid domestic environments such as kitchens, bathrooms and domestic appliances, containing or operating with water. In this review, we will therefore mainly focus on this subject, limiting the overview of air-borne, dust-associated microbiome research to a brief summary.

Indoor dust includes fungal conidia and spores, spore fragments, dead and living bacterial cells, their fragments, endospores and other spores. Thus, up to 500–1000 different species can be present in house dust (Rintala et al. 2012). Culture-independent methods indicate that the house dust bacterial community is dominated by Gram-positive bacteria, especially *Firmicutes* and *Actinobacteria*. These bacteria originate primarily from human-associated sources (Hanson et al. 2016; Noris et al. 2011; Rintala et al. 2008; Taubel et al. 2009). Fungi can also be present in dust in great numbers. Thus, standard fungal cultivation methods showed up to 70 million CFU/g of dust (Beguin and Nolard 1996). Such dust-associated fungi are typically related to the outside environment (Shan et al. 2019). Although the most cosmopolitan fungal taxa are common everywhere, the latitude and season influence their indoor diversity. In general, these dust-related fungal communities are more diverse in comparison with the bacterial indoor biome (Dannemiller et al. 2016). The composition of these indoor microbial communities is associated with the levels of urbanisation and the health of the inhabitants, in particular concerning allergic disorders, intestinal microbiome and general immune responses (Shan et al. 2019).

Water availability is a major requirement for microbial growth. Indoor water-borne microorganisms are not confined only to bathrooms and kitchens but can also populate tap water systems and household appliances, in which they can be attracted to certain chemicals and plastic or rubber surfaces. These conditions are not conductive to growth of most microorganisms; however, some adapted microbes, and in particular fungi, are able to metabolise phenols and hydrocarbons (Prenafeta-Boldu et al. 2006) and are generally very adaptable, resilient and in some cases also thermotolerant (Gostinčar et al. 2011, 2015; Zalar et al. 2011; Zupančič et al. 2016). Additionally, as a side effect of attempts to improve the energy-efficiency and environmental friendliness of household appliances, these became more prone to microbial growth. Already in 1999, Beadle and Verran stated that lower temperatures for washing and less aggressive detergents without bleach became commonly used (Beadle and Verran 1999). While becoming more permissive, these conditions are still harsh enough to select for the more stress-resistant, but also often, virulent microbes, with greater potential to cause opportunistic human infections (Byrd-Bredbenner et al. 2013; Callewaert et al. 2015; Döğen et al. 2013a, b; Dunn et al. 2013; Gümral et al. 2016; Gostinčar et al. 2011, 2015; Raghupathi et al. 2018; Rehberg et al. 2017; Sasahara et al. 2011; Ståhl Wernersson et al. 2004; Zalar et al. 2011; Zupančič et al. 2016, 2018).

Research on water-related indoor microorganisms so far mostly focused on bacteria important for human health (Larsen et al. 2010). Over the last 10 years, however, it became apparent that fungi occupy several wet or humid domestic niches exemplified by home appliances such as dishwashers and washing machines. The diversity of these fungal “water-biomes” turned out to be dominated by polyextremotolerant, oligotrophic species, some of which are opportunistic human pathogens, representing a potential health problem that became widely recognised only in the last years (Zalar et al. 2011; Hamada and Abe 2010; Lian and de Hoog 2010; Defra 2011; Fouquier et al. 2016). Therefore, this review will focus in particular on the diversity of polyextremotolerant fungi in domestic wet environments, and some evolutionary aspects of their adaptive abilities as well as their pathogenic potential.

## Water as a transmission vector of indoor microorganisms

Before being suitable for water consumption, raw water originating from either surface water or groundwater is subjected to a variety of physico-chemical treatments in order to remove larger particles and microorganisms. The efficiency of removing microbes from raw water greatly depends on the combination of the cleaning techniques used and may vary between 8 and 99.99% (WHO 2011). Water for human consumption should not contain potentially harmful microorganisms or dangerous substances, making it necessary to control not only the input water quality, but also the formation of biofilms and increases of the microbial load in the distribution system. Global requirements, directives and recommendations, which are the basis for other acts and standards, are currently directed by the World Health Organisation (WHO). Since WHO follows microbiological trends and health problems on a global level, they list a large spectrum of microbial genera that are important for monitoring water quality and thus ensuring public health (WHO 2011). The mandatory microbiological parameters used for the determination of water quality in the EU are the presence of the bacteria *Escherichia coli*, *Clostridium perfringens*, *Pseudomonas aeruginosa*, coliforms and the total number of detectable aerobic microorganisms after incubation at 22 °C and 37 °C (EEC 1998). Enteric viruses and occurrence of oocysts and cysts belonging to the protozoa *Giardia lamblia, Cryptosporidium* spp. and *Entamoeba hystolitica* can also be used as additional indicators of water quality, recommended by WHO (WHO 2011). Similarly within the EU, the United States Environmental Protection Agency (US EPA) issued the National Primary Drinking Water Regulations, but with some differences from the EU Drinking Water Directive 98/83/EC. For example, US EPA determines the limits for heterotrophic plate count, total coliforms, *Cryptosporidium*, *Giardia lamblia*, *Legionella, Mycobacterium* and enteric viruses (US EPA 2009, 2018).

## Eukaryotic (micro) organisms in drinking water are often neglected

Despite routine monitoring of several microbial parameters in water, these often do not cover spore-forming microorganisms and microbes aggregating in clumps. This can be seen as problematic, since such microorganisms are typically more resistant to the water treatment process and enter households via plumbing systems, where they are the cause of significant problems related to accumulation of biofilms and corrosion of materials. If present in large quantities, they also change the odour of water and may affect human health via toxin production (Novak Babič et al. 2017a, b).

Water is the main transmission medium of algae. Due to the need for light, algae can be found in the upper layers of natural water sources, particularly in lakes and rivers with low water flow (Wurzbacher et al. 2011). Some algal species belonging to the genera *Scenedesmus*, *Euglena*, *Anacystis*, *Coelastrum*, *Prototheca* and *Chlorococcum* can be found in tap water systems, despite chlorination, and can survive without light (Defra 2011)*.* They contribute to the primary production of oxygen and biomass representing food for other microorganisms. Algae in tap water systems are particularly problematic as reservoirs of certain bacteria (e.g. *Legionella*). This phenomenon has been observed in genera *Elakatothrix*, *Gomphosphaeria*, *Closterium*, *Cosmarium* and *Chlorella* (Defra 2011). According to the WHO, algae producing toxins, and those causing bad taste or odour, should be absent from tap water (WHO 2011).

Water is the main transmission medium not only of algae but also of protists. Protists, particularly species from the genera *Acanthamoeba*, *Naegleria*, *Saccamoeba*, *Hartmannella* and *Vexillifera*, control the number of bacteria and to a lesser extent also of fungi (Defra 2011; Percival et al. 2014). Like algae, some represent hosts for pathogenic bacteria of the genera *Legionella*, *Burkholderia* and *Mycobacterium*. Additionally, species from the genera *Cryptosporidium*, *Microsporidia* and *Giardia* can act as human pathogens, causing diarrhoea, nausea, keratitis and encephalitis. Species that can form oocysts represent particular problems, since they are less sensitive to water chlorination (Valster 2011). According to the WHO *Acanthamoeba* spp., *Cryptosporidium hominis*, *C. parvum*, *Cyclospora cayetanensis*, *E. histolytica*, *Giardia intestinalis* and *Naegleria fowleri* should be absent from water. Monitoring of *Balantidium coli*, *Blastocystis hominis*, *Isospora belli*, *Toxoplasma gondii* and Microsporidia is also suggested (WHO 2011).

The least investigated group of eukaryotes with poorly understood roles in the water microbiome are fungi. Some species such as *Candida parapsilosis*, *Penicillium* spp. and *Aspergillus* spp. can represent a scaffold for microbial attachment (Zupančič et al. 2019; Richardson and Rautemaa-Richardson 2019; Fernandes et al. 2019) and food for protozoa (Defra 2011). Mainly, filamentous fungi also contribute to the formation of biofilms by cross-linking particulate matter and microbial polymers with hyphae (Richardson and Rautemaa-Richardson 2019; Fernandes et al. 2019). Biofilms in tap water systems may contain up to 8.9 CFU/cm^2^ of yeasts and 4.0–25.2 CFU/cm^2^ of filamentous fungi (Doggett 2000). A great variety of fungi was detected in drinking water either by culture-dependent or molecular methods (e.g. with metagenome of amplicon sequencing). Their diversity significantly depends on the source of raw water (Moat et al. 2016; Novak Babič et al. 2017a, b). Water from surface water sources is dominated by plant-degrading fungi of the genera *Acremonium*, *Altenaria*, *Arthrinium*, *Aspergillus*, *Beauveria*, *Botrytis*, *Cladosporium*, *Cryptococcus*, *Epicoccum*, *Fusarium*, *Geotrichum*, *Gliocladium*, *Mucor*, *Paecilomyces*, *Penicillium*, *Phoma*, *Rhizopus*, *Rhodotorula*, *Sporothrix* and *Trichoderma*. Yeasts *Candida* spp., *Cystobasidium* spp. and black yeast-like fungi *Aureobasidium*, *Cladophialophora*, *Cyphellophora*, *Exophiala*, *Phialophora* and *Rhinocladiella* are more often found in water originating from the groundwater (Novak Babič et al. 2017a, b). Due to the lack of information linking clinical infections and the fungal presence in drinking water, fungi are not yet considered an independent parameter in water regulations. However, their presence in drinking water can alter the taste and odour of water, while in higher numbers they can impair human health causing allergies, asthma and superficial infections of hair, skin and nails (Defra 2011; Novak Babič et al. 2017a, b).

## Bacteria in water microbiome—still a great mystery?

Bacteria are the most numerous microorganisms in tap water and biofilms of the water distribution system. Since the 18th century, people are aware of transmission of faecal bacteria via the plumbing system (Percival et al. 2014). In inappropriately treated water sources, after natural disasters or due to faults in water supply systems, the number of pathogenic bacteria involved in infections of the digestive and respiratory tract can exceed the prescribed safe limits (Percival et al. 2014). The presence of causative agents of gastrointestinal illnesses thus needs to be monitored regularly. The most problematic bacteria potentially found in tap water according to WHO are *Vibrio cholerae*, *Salmonella typhi*, *Shigella* spp., *Campylobacter jejuni*, *E. coli*, *Yersinia enterolitica*, *Legionella* spp., *Aeromonas* spp., *Mycobacterium* spp., *Bacillus* spp. and *P. aeruginosa* (WHO 2011; EEC 1998). Caution is also needed in case of rapid propagation of toxin-producing Cyanobacteria of the genera *Microcystis*, *Planktothrix*, *Anabaena*, *Oscillatoria* and *Aphanizomenon* (Szewzyk et al. 2000; Percival et al. 2014). However, the presence, diversity and ecological interactions of water-borne bacteria remain poorly investigated and understood. These gaps in knowledge are now primarily addressed using high-throughput sequencing methods (Callewaert et al. 2015). The number of bacteria reaches its lowest limits in water tanks after treatment procedures (e.g. chlorination), while in tap water the number of bacteria increases again due to the presence of biofilms in plumbing systems (Kormas et al. 2010). Thus, the numbers of CFU in groundwater can be up to 10^6^ CFU/mL, in well water up to 10^3^ CFU/mL, in reservoir tanks after chlorination up to 10^2^ CFU/mL and in household tap water between 10^2^ and 10^5^ CFU/mL (Kormas et al. 2010; Callewaert et al. 2015). Their diversity depends greatly on environmental factors such as disinfectant, temperature, flow and building materials (Wang et al. 2014). Typical bacteria in ground and well water are *Betaproteobacteria* (Kormas et al. 2010). The same class is usually also dominant in cold water in the indoor plumbing system (Inkinen et al. 2016). In biofilms of the lower parts of the plumbing system (e.g. basement and first floor), in systems with regular water consumption and relatively high water flow, *Alphaproteobacteria* dominate over *Betaproteobacteria* (Inkinen et al. 2016). This is also true in copper pipes (Inkinen et al. 2016), while cross-linked polyethylene (PEX) pipes in combination with hot water appear to select for *Betaproteobacteria*, *Gammaproteobacteria*, *Actinobacteria* and *Bacilli* (Callewaert et al. 2015; Inkinen et al. 2016; Moat et al. 2016).

## Indoor biomes are shaped by selection driven by anthropogenic factors

**Fig. 1:**
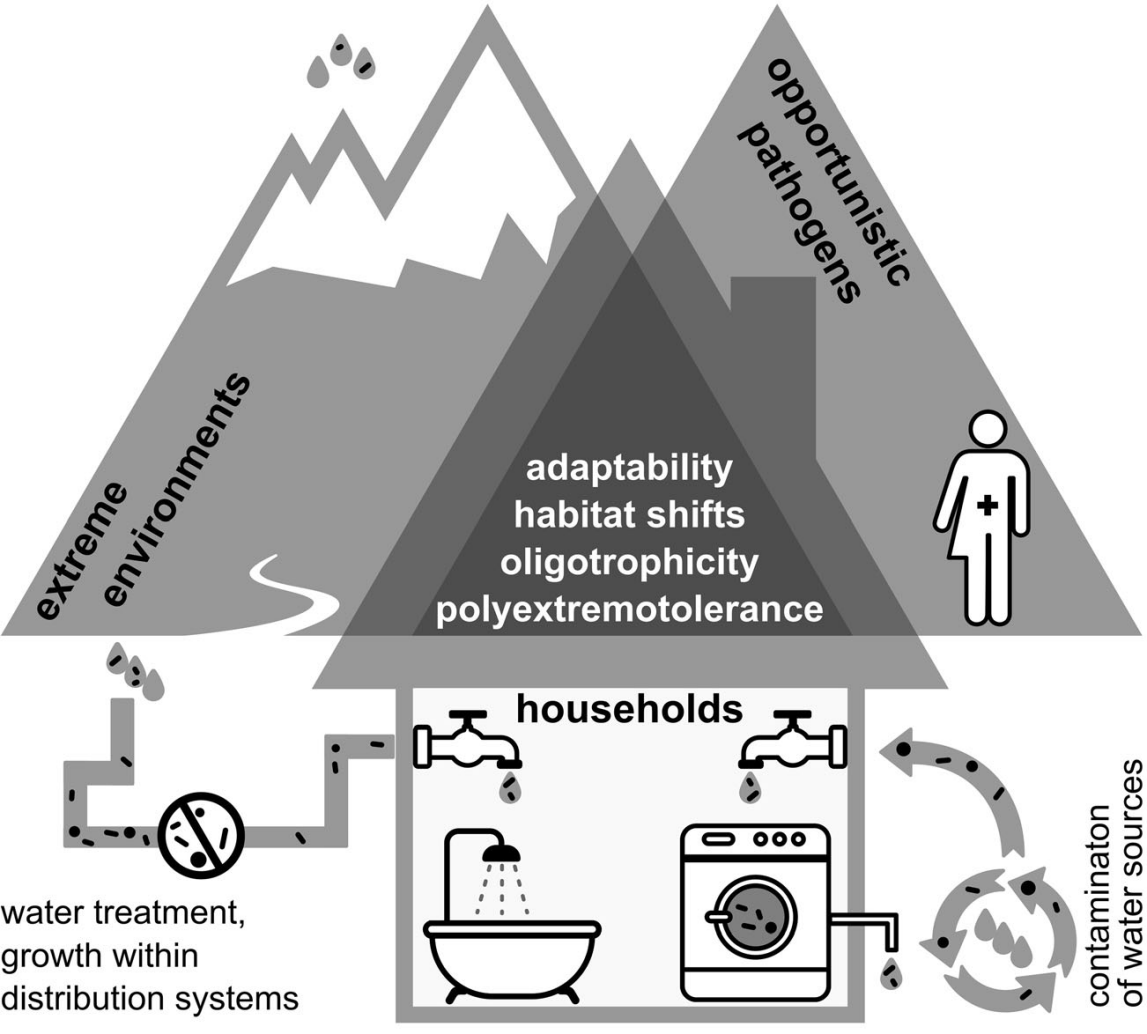
Water-related microorganisms in the households. Biofilm formation, oligotrophicity, resistance to water treatment procedures, resistance to occasional desiccation and cleaning procedures are among traits promoting survival of water-related microbes indoors. Species surviving such conditions are characterised by great adaptability, stress tolerance and capability of habitat shifts. All of these are traits they share with some species found in extreme natural environments and also in opportunistic pathogens, for which the infection of the human body can be seen as just another habitat shift exposing them to a new and stressful environment. Tap water distribution systems provide opportunities for microbial growth of adapted microorganisms. After entering the household with water, different habitats support the formation of many, in some cases very specific communities, shaped by the conditions of each habitat (such as shower heads or household appliances). In some cases, the enriched microorganisms leave the household in large numbers (e.g. with wastewater), possibly contaminating water sources and re-entering the households at a later time

Microorganisms entering buildings via tap water become exposed to different physico-chemical factors, which shape the diversity of the communities in indoor habitats. Exposure to unfavourable life conditions (e.g. desiccation, high or low temperatures, variable pH) leads to a constant selection of the best adapted species. A metagenomic study of indoor microorganisms reported enrichment of genes involved in overcoming iron limitation, oxidative damage and desiccation (Tringe et al. 2008). While high humidity makes some indoor habitats more prone to microbial growth, even the most humid parts of the household represent major challenges to microorganisms. Not only because water availability is often not constant (occasional desiccation triggers high levels of stress), but also due to other conditions. These include the often scarce sources of nutrients, which have to be derived from the environment or from the building materials (Gorny 2004), sometimes including unusual compounds such as surfactants found in cosmetic products (Hamada and Abe 2010) or from other microbes. Studies of microbial diversity of indoor environments have shown that indoors should not be seen as a homogenous environment. Instead, many different habitats can be recognised, some very specific, with profoundly different microbial communities, shaped by different selection pressures. Hostile conditions usually also result in a rather limited number of species found in each specific habitat. Showerheads, with their abundance of water, represent a challenge to microbes due to mechanical forces and water chlorination, two factors that presumably select for biofilm-forming species (Feazel et al. 2009) (Fig. 1). As will be discussed later, the drive towards the use of biodegradable materials, including the substitution of aggressive chemical detergents with more environmentally friendly alternatives, not only decreases the amount of antimicrobial compounds in the household, but in some cases even provides additional nutrients. This does not mean that antimicrobial substances do not come with their own set of potential problems. One of the infamous antibacterial compounds used and released in great quantities is triclosan (Levy 2001; Fang et al. 2010), which is used not only despite that fact that its benefits remain unproven (FDA 2019), but also despite the risks it poses—antimicrobials such as triclosan have for example been linked to the selection of bacteria with higher resistance to antibiotics (Westfall et al. 2019). Other factors that limit microbial diversity and abundance indoors are oxidative stress (oxidative chemicals, such as bleach or hydrogen peroxide, are found in many household cleaning agents), chemicals that change chaotropicity (Hallsworth et al. 2003) or hydrophobicity (Bhaganna et al. 2010), and provide unusual sources of nutrients, such as aromatic pollutants.

Microbial growth in indoor environments is difficult to overlook and at least bacterial abundance has been studied for decades, focusing particularly on the places most prone to contamination, such as kitchens and bathrooms (Ojima et al. 2002; Beumer and Kusumaningrum 2003). Compared to bacteria, the high abundance of fungi in anthropogenic habitats is less studied. Some recent studies have uncovered a surprising diversity including pathogenic species in hospitals (Garcia-Cruz et al. 2012). Opportunistic fungal pathogens are present also in residential buildings, where habitats with regular presence of liquid water (e.g. plumbing, sinks or drains) support active growth and accumulation of high biomass, while dry surfaces contain mostly species deposited from air and aerosols (Adams et al. 2013; Zalar et al. 2011). Polyextremotolerant oligotrophic fungi are particularly common in indoor wet cells (Zalar et al. 2011; Hamada and Abe 2010; Lian and de Hoog 2010).

## Indoor environments dominated by water-borne microorganisms

### Kitchen surfaces

Among places inside our homes, kitchens are most heavily colonised by microbes and are a place where people are typically exposed to the broadest diversity of microorganisms while indoors (Sinclair and Gerba 2011). Exposure to microbes comes from handling and preparing food and from contact with kitchen surfaces that harbour microorganisms derived from food, water, air and humans (Medrano-Felix et al. 2011). Although outdoor and indoor air and water are known sources of microbial contamination of kitchens, human skin was found to be the primary source of bacteria belonging to the families *Propionibacteriaceae*, *Corynebacteriaceae*, *Staphylococcaceae* and *Streptococcaceae* (Flores et al. 2011; Flores et al. 2012). Diversity of bacterial communities of typical household kitchens was studied in detail only recently and it revealed that moist surfaces in kitchens usually yield the highest number of microbes due to the presence of water essential for microbial propagation (Sinclair and Gerba 2011; Flores et al. 2012). Most abundant bacteria belong to the families *Micrococcaceae*, *Flavobacteriaceae*, *Streptococcaceae* and *Moraxellaceae* (Flores et al. 2012). These families contain bacterial genera able to survive on solid surfaces for a long time (Santo et al. 2010). Most diverse bacterial communities were found outside the stove exhaust fans and on the surface of the floor as a consequence of passive and active deposition of particulates and infrequent cleaning. On the other hand, the least diverse communities were observed on metallic surfaces (kitchen sinks, faucets, sink drains, sink basins) and were dominated by Gram-negative bacteria, known to produce stable biofilms (e.g. *Sphingomonadaceae*) (Kelley et al. 2004; Flores et al. 2012). Gram-positive *Firmicutes*, able to form spores, were more abundant on dry surfaces (e.g. floors, cabinets and microwaves) (Flores et al. 2012). While the majority of bacteria found on kitchen surfaces are harmless, some surfaces with a direct contact with raw food can harbour pathogenic bacteria (Berger et al. 2010). In developed countries, bacterial food-borne illnesses are mainly connected to different bacterial species *Bacillus cereus*, *Campylobacter* spp., *Salmonella* spp., *C. perfringens*, *E. coli* and *Listeria monocytogenes* (Mead et al. 1999; Bintsis 2017). *Campylobacter* species were mainly present on surfaces above counter tops, on upper cabinet handles and on the microwave panels. Contamination of these surfaces is thought to occur via hands of persons who handled raw poultry, known to be frequently contaminated with *Campylobacter* (Luber 2009; Flores et al. 2012).

Kitchen sponges are the most colonised object in kitchens, with the highest load of microbes. Their porous construction, constantly humid environment and contact with food enable successful colonisation and propagation of microbes (Alwakeel 2007). Bacteria *E. coli*, *Enterobacter cloacae*, *Proteus* spp., *Klebsiella* spp., *Campylobacter* spp. and *Salmonella* spp. usually originating from food ingredients are commonly present on kitchen sponges and later transferred to the surfaces made of steel or plastics (Rossi et al. 2013; Cardinale et al. 2017). Next-generation sequencing (NGS) also revealed the presence of water-borne bacteria *Acinetobacter johnsonii*, *A. pittii*, *A. ursingii*, *Chryseobacterium hominis* and *Moraxella osloensis* belonging to the Biosafety Level 2 along with known biofilm producers of the genera *Pseudomonas*, *Sphingomonas* and *Rhizobium* (Table 1) (Cardinale et al. 2017). Among fungi, genus *Aspergillus* is the most commonly encountered (Alwakeel 2007).

**Table 1:** The most common bacterial and fungal species reported from different water-related indoor habitats

Indoor habitat	Bacteria	Fungi	References
Kitchens	*Acinetobacter johnsonii* *Acinetobacter pittii* *Acinetobacter ursingii* *Bacillus cereus* *Campylobacter* spp. *Clostridium perfringens* *Chryseobacterium hominis* *Enterobacter cloacae* *Escherichia coli* *Klebsiella* spp. *Listeria monocytogenes* *Moraxella osloensis* *Proteus* spp. *Pseudomonas* spp. *Rhizobium* spp. *Salmonella* spp. *Sphingomonas* spp.	*Apiotrichum* spp. *Aspergillus* spp. *Aureobasidium* spp. *Candida* spp. *Capronia* spp. *Cladophialophora* spp. *Cladosporium* spp. *Cryptococcus* spp. *Cyphellophora* spp. *Cystobasidium* spp. *Exophiala* spp. *Fonsecaea* spp. *Fusarium* spp. *Hyphopichia* spp. *Issatchenkia* spp. *Knufia* spp. *Malassezia* spp. *Meyerozyma* spp. *Naganishia* spp. *Ochroconis* spp. *Penicillium* spp. *Phoma* spp. *Pichia* spp. *Phialophora* spp. *Rhinocladiella* spp. *Rhodotorula* spp. *Trichosporon* spp. *Wickerhamiella* spp. *Wickerhamomyces* spp.	Adams et al. 2013 Alwakeel 2007 Bintsis 2017 Cardinale et al. 2017 Flores et al. 2011 Flores et al. 2012 Kelley et al. 2004 Mead et al. 1999 Novak Babič et al. 2016b Rossi et al. 2013 Wang et al. 2018 Zupančič et al. 2016
Dishwashers	*Acinetobacter baumannii* *Bacillus cereus* *Escherichia coli* *Pseudomonas aeruginosa Stenotrophomonas maltophilia*	*Candida parapsilosis* *Exophiala dermatitidis* *Exophiala phaeomuriformis* *Fusarium* spp. *Magnusiomyces capitatus* *Meyerozyma guilliermondii* *Pichia* spp. *Rhodotorula mucilaginosa* *Saccharomyces cerevisiae* *Saprochaete clavata*	Döğen et al. 2013a, b Gümral et al. 2015 Zalar et al. 2011 Zupančič et al. 2016 Zupančič et al. 2019
Refrigerators and freezers	*Arthrobacter* spp. *Bacillus* spp. *Carnobacterium* spp. *Escherichia coli* *Flavobacterium* spp. *Lactobacillus* spp. *Listeria* spp. *Pantoea* spp. *Pseudomonas* spp. *Salmonella* spp. *Sphingobacterium* spp. *Staphylococcus* spp.	*Acremonium* spp. *Alternaria* spp. *Aspergillus* spp. *Botrytis cinerea* *Candida* spp. *Cladosporium* spp. *Cyphellophora* spp. *Exophiala* spp. *Knufia* spp. *Mucor racemosus* *Ochroconis* spp. *Penicillium italicum* *Rhinocladiella* spp. *Rhizopus oryzae* *Scopulariopsis* spp.	Altunatmaz et al. 2012 Buchholz et al. 2011 Jeon et al. 2013 Maktabi et al. 2013 Wang et al. 2018
Bathrooms	*Citrobacter* spp. *Enterobacter* spp. *Enterococcus* spp. *Escherichia* spp. *Klebsiella* spp. *Legionella* spp. *Mycobacterium* spp. *Pseudomonas* spp. *Serratia* spp. *Streptoccous mutans* *Streptococcus* spp. *Staphylococcus* spp.	*Aspergillus* spp. *Aureobasidium* spp. *Candida* spp. *Cladosporium* spp. *Cryptococcus* spp. *Cyphellophora* spp. *Exophiala* spp. *Fusarium* spp. *Knufia* spp. *Leptosphaeria* spp. *Malassezia* spp. *Naganishia* spp. *Ochroconis* spp. *Penicillium* spp. *Phialophora* spp. *Rhinocladiella* spp. *Rhodotorula* spp. *Schizophylum* spp.	Ankola et al. 2009 Cole et al. 2003 Eguchi et al. 2013 Hamada and Abe 2010 Hamada and Fujita 2000 Moat et al. 2016 Novak Babič et al. 2017a, b Novak Babič et al. 2017b Wang et al. 2018
Washing machines	*Acinetobacter* spp. *Bacillus* spp. *Clostridium difficile* *Corynebacterium* spp. *Enhydrobacter* spp. *Escherichia coli* *Klebsiella pneumoniae* *Klebsiella oxytoca* *Micrococcus luteus* *Pseudomonas* spp. *Sphingomonas* spp. *Staphylococcus aureus*	*Alternaria* spp. *Aspergillus* spp. *Candida albicans* *Candida parapsilosis* *Capronia* spp. *Cladosporium* spp. *Cryptococcus* spp. *Cystobasidium slooffiae* *Exophiala* spp. *Fusarium oxysporum* species complex (FOSC) *Knufia* spp. *Microsporum canis* *Naganishia* spp. *Ochroconis* spp. *Penicillium* spp. *Rhodotorula* spp. *Trichophyton* spp. *Trichosporon* spp.	Callewaert et al. 2015 Gattlen et al. 2010 Kubota et al. 2012 Novak Babič et al. 2015 Panagea et al. 2005 Perry et al. 2001 Rozman et al. 2013 Schmithausen et al. 2019 Stapleton et al. 2013 Tanaka et al. 2006 Wang et al. 2018
Clothes dryers	*Bacillus* spp. *Staphylococcus aureus* *Streptococcus pyogenes*	No data	Brunton 1995 Fijan and Šostar Turk 2012
Saunas	*Bacillus* spp. *Janthinobacterium* sp. *Pseudoxanthomonas taiwanensis* *Stenotrophomonas* sp. *Tepidomonas* sp. *Virgibacillus* sp.	*Candida* spp. *Exophiala dermatitidis* *Phialophora* spp. *Rhinocladiella* spp.	Lee and Park 2012 Novak Babič et al. 2017b

**Fig. 2:**
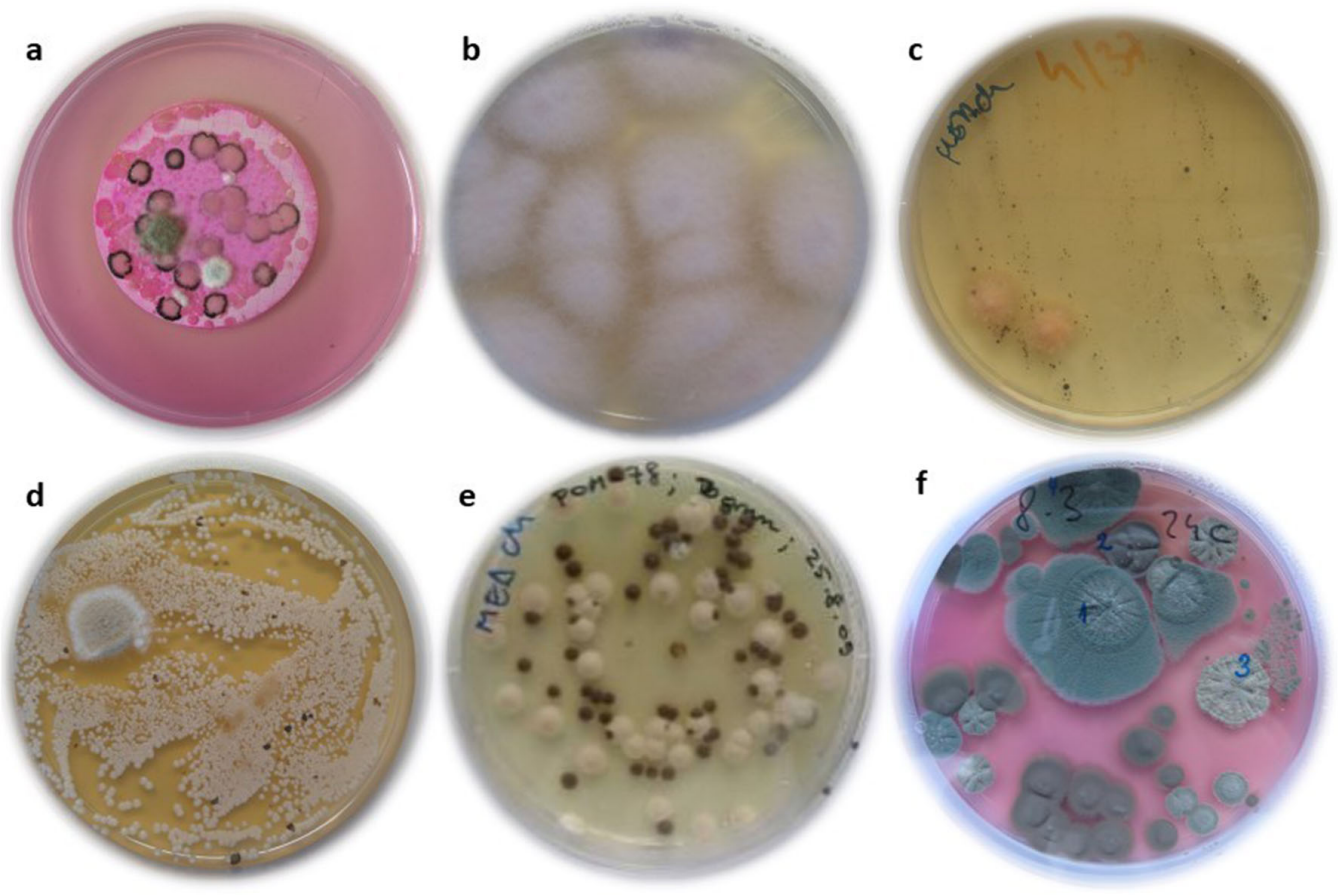
Isolation plates with fungi from different indoor habitats linked to water. **a** Filter paper covered with colonies of *Aureobasidium melanogenum* and *Aspergillus* spp. isolated from 1 l of drinking water on Dichloran Rose Bengal Agar with addition of chloramphenicol (DRBC+Ch). **b***Fusarium oxysporum* species complex (FOSC) isolated on Malt Extract Agar with addition of chloramphenicol (MEA+Ch). Swab sample was taken from a detergent drawer in a washing machine. **c** Black yeasts and orange colonies of *Bisifusarium dimerum* isolated on Malt Extract Agar with addition of chloramphenicol (MEA+Ch). Swab sample was taken from a silicone in the bathroom. **d** Colonies of *Candida parapsilosis* sensu stricto and the black yeast *Exophiala phaeomuriformis* isolated on Malt Extract Agar with addition of chloramphenicol (MEA+Ch). Swab sample was taken from moist kitchen desk. **e** Colonies of *C. parapsilosis* sensu stricto and the black yeast *E. dermatitidis* isolated on Malt Extract Agar with addition of chloramphenicol (MEA+Ch). Swab sample was taken from the rubber seal on the dishwasher’s door. **f** Colonies of *Aspergillus* spp. and *Cladosporium* spp. isolated on Dichloran Rose Bengal Agar with addition of chloramphenicol (DRBC+Ch). Swab sample was taken from the rubber seal on the refrigerator’s door

Fungal presence in household kitchens was for a long time connected only to indoor air and dust, or contaminated food. Recently, conducted studies suggested tap water as an important additional vector (Novak Babič et al. 2016b). Fungal presence and active growth were recently investigated on sills, drains, sinks and pipes (Adams et al. 2013; Zupančič et al. 2016) (Fig. 2). Ubiquitous genera of filamentous fungi like *Aspergillus*, *Cladosporium* and *Penicillium* were present mainly on surfaces exposed to outdoor air (e.g. sills next to windows) (Adams et al. 2013), while moist surfaces in regular contact with water harboured yeasts from the genera *Apiotrichum*, *Candida*, *Cryptococcus*, *Cystobasidium*, *Issatchenkia*, *Malassezia, Meyerozyma*, *Naganishia*, *Hyphopichia*, *Pichia*, *Rhodotorula*, *Trichosporon*, *Wickerhamiella* and *Wickerhamomyces*, and black yeasts *Aureobasidium*, *Capronia*, *Cyphellophora*, *Cladophialophora*, *Exophiala*, *Fonsecaea*, *Knufia*, *Phialophora* and *Rhinocladiella* (Table 1) (Zupančič et al. 2016; Wang et al. 2018), many of which were previously reported also from tap water (Zupančič et al. 2016; Novak Babič et al. 2016b). Filamentous fungi belonging to the genera *Fusarium*, *Ochroconis* and *Phoma* were also regularly encountered, especially in biofilms on drains and sinks (Adams et al. 2013; Zupančič et al. 2016). Species that should cause the most concern are human opportunistic fungi, able to cause diseases in immunocompromised people, particularly species secreting extracellular polysaccharides (EPS). These species can form mixed bacterial-fungal biofilms and can thus adhere more easily to human hands (e.g. species from genera *Candida*, *Cryptococcus*, *Malassezia* and *Rhodotorula*). Fungi spread by air or aerosols, such as representatives of the genera *Aspergillus*, *Cladosporium*, *Fusarium* and *Penicillium*, can pose a risk for people suffering from allergies or asthma. Some fungi can present problems if inoculated on the skin or subcutaneously by skin trauma, melanised black fungi being particularly problematic (de Hoog et al. 2014).

### Bathrooms

Bacteria colonising bathroom surfaces have various routes of entry. The major ones are tap water, human skin and oral biota, and faecal contamination (in case of coliform bacteria) (Ankola et al. 2009). Water-related bacteria are colonising interiors of water pipes and shower hoses and belong mainly to *Alphaproteobacteria* followed by *Actinobacteria* (Moat et al. 2016). Pathogens of the genera *Pseudomonas*, *Mycobacterium* and *Legionella* are commonly encountered (Moat et al. 2016). These are dispersed through aerosols and are able to cause respiratory infections. Other infections are also possible, such as the *P. aeruginosa* keratitis due to the biofilm formation on contact lenses left in bathroom, reported by Eguchi et al. (2013).

After kitchen sponges, the object with the second highest microbial load indoors are toothbrushes. Similarly, these are made of a polymer with a large surface, which is constantly wet and repeatedly exposed to microbes present in the oral cavity and on the skin (Patcas et al. 2018). Bacteria *Streptoccous mutans*, β-haemolytic streptococci and yeast *C. albicans* are the most common residents of plastic bristles and causative agents of repeated infections (Ankola et al. 2009). Other skin- and faeces-related bacteria, such as those from the genera *Citrobacter*, *Enterobacter*, *Escherichia*, *Klebsiella*, *Serratia*, *Enterococcus*, *Streptococcus* and *Staphylococcus* spp. could be found on the floor, drains, sinks, toilet seats and counters across the bathrooms (Table 1) (Cole et al. 2003).

Similarly to bacteria, fungi colonising bathroom surfaces can also originate from tap water or skin. Water-borne fungi represent a diversity of species from the genera *Aureobasidium*, *Cladosporium*, *Cryptococcus*, *Cyphellophora*, *Exophiala*, *Fusarium*, *Knufia*, *Leptosphaeria*, *Malassezia*, *Naganishia*, *Ochroconis*, *Phialophora*, *Rhinocladiella* and *Schizophylum* (Table 1) (Wang et al. 2018; Novak Babič et al. 2017a, b; Moat et al. 2016). These genera can be isolated from taps, sinks and seals between ceramic bathroom tiles (Fig. 2) (Wang et al. 2018). Shower curtains, sinks and floors in contact with skin are additionally colonised with *Candida* and *Rhodotorula* (Hamada and Fujita 2000; Novak Babič et al. 2017b). Use of soaps and shampoos provides the scaffold for biofilm formation and greatly supports the growth of alkali-tolerant fungi degrading the ingredients of detergents (Hamada and Fujita 2000; Hamada and Abe 2010; Lian and de Hoog 2010). Constantly humid conditions also support the growth of air-borne fungi of the genera *Aspergillus*, *Cladosporium* and *Penicillium*, which often colonise upper walls and ceilings (Hamada and Fujita 2000; Hamada and Abe 2010; Wang et al. 2018).

### Saunas

Saunas usually harbour spore-forming bacteria, able to sustain high temperatures, pressure and humidity. The most commonly isolated bacterial species thus belonged to the genus *Bacillus*. Other bacteria were identified as *Virgibacillus* sp., *Tepidomonas* sp., *Pseudoxanthomonas taiwanensis*, *Stenotrophomonas* sp. and *Janthinobacterium* sp. (Lee and Park 2012). Similar trends can be observed for fungi, where more resilient thermotolerant and melanised species, such as *Exophiala dermatitidis*, *Phialophora* and *Rhinocladiella*, often dominate over other yeasts, such as *Candida* and dermatophytes (Table 1) (Novak Babič et al. 2017b).

## Household appliances enrich extremophilic microorganisms

### Dishwashers

In the last few decades, the presence of household dishwashers became widespread. This created a new habitat for harbouring and spreading potentially pathogenic microbes. Because of relatively high temperatures during washing cycles, regular addition of detergents and steel interior, it was believed that microbes can be completely removed and that any microbial presence inside the dishwasher was only transient and decidedly unproblematic. However, the first systematic study reported a consistent presence of certain species of fungi, including opportunistic pathogens, on rubber seals worldwide (Zalar et al. 2011). Dishwasher seals were colonised with different species regardless of their age, brand mark or use of detergents. Surprisingly, some fungi that are only rarely recovered from environment appeared to be main colonisers of dishwashers. Among them, opportunistic black yeasts *E. dermatitidis* and *E. phaeomuriformis* were found in the highest numbers, colonising between 30 and 50% of sampled dishwashers. Studies also revealed the presence of different *Candida* species, *C. parapsilosis* being predominant (Fig. 2), as well as the presence of otherwise ubiquitous *Rhodotorula mucilaginosa* and *Fusarium* spp. (Table 1) (Zalar et al. 2011; Döğen et al. 2013a, b; Gümral et al. 2015). In the following studies, results confirmed the existence of a core mycobiota (Zupančič et al. 2016) and identified tap water as the main source of contamination of dishwashers with these fungi (Novak Babič et al. 2016b). To a lesser extent, food also contributes to the fungal load in dishwashers, particularly with species *Saccharomyces cerevisiae*, *Pichia* spp. and opportunistic pathogens from the genera *Magnusiomyces* and *Saprochaete* (Zalar et al. 2011; Zupančič et al. 2016). Dishwashers not only represent a novel habitat, but also appear to act as a source of contamination. The presence of dishwashers has an important impact on the presence of opportunistic fungi on different kitchen surfaces. Kitchens without dishwashers are mainly colonised with species of the genera *Candida, Meyerozyma, Pichia* and *Rhodotorula*, while kitchens with dishwashers have significantly higher numbers of black yeasts from the genus *Exophiala*. It was confirmed that fungi from dishwashers can be present in hot aerosols, released immediately at the end of the washing cycle and in wastewater drained from the dishwashers (Zupančič et al. 2016).

The medical importance of opportunistic fungi colonising dishwashers is not well understood. So far, only one case report linked the infections with an opportunistic fungus and contaminated dishwashers. Menu et al. (2019) reported outbreaks caused by *Saprochaete clavata* in a haematology unit in Marseille, France. Genotyping later confirmed the same origin of the strains present in dishwashers used by the patients and their families and the clinical isolates of the fungus (Menu et al. 2019).

The presence of bacteria in dishwashers was investigated later than the presence of fungi. A study conducted on 30 dishwasher rubber seals in Slovenia (Zupančič et al. 2019) revealed the presence of 74 species of bacteria. The majority were Gram-positive and sporogenic. Among them, bacteria from the phyla *Firmicutes* and *Actinobacteria* prevailed, with *Bacillus cereus* group being the most abundant. The study found several potentially pathogenic species, including *Acinetobacter baumannii*, *P. aeruginosa* and *Stenotrophomonas maltophilia* (Table 1)*.* Strains of *E. coli* belonged to a non-pathogenic phylogenetic group A. The most tested bacteria showed high resistance to antibiotics such as cefotaxime, ceftazidime and ertapenem, while genes for virulence markers were detected only in *P. aeruginosa* strains. To evaluate water as the possible vector of contamination of dishwashers with bacteria, a metagenomic analysis of bacterial diversity in tap water was conducted. The study showed the dominance of *Alphaproteobacteria* and *Betaproteobacteria* in the water, while *Actinobacteria* and *Firmicutes* were present in much lower numbers. These results indicate that food and used dishes are the main source of bacteria found in dishwashers (Zupančič et al. 2019).

### Refrigerators and freezers

Refrigerators and freezers operate at temperatures between − 18 and + 12 °C. These temperatures slow down the metabolism of mesophilic microbes; however, they enable the growth of psychrotolerant and psychrophilic microorganisms. Additionally, differences in temperature inside appliances result in condensate formation and thus a relatively constant source of water, while parts made of plastic and rubber facilitate microbial attachment and biofilm formation. It was shown that the concentration of viable cells in refrigerators can be up to 10^7^ CFU/cm^2^ (Kennedy et al. 2005). Inner surfaces of refrigerators and freezers, especially door seals, are cleaned infrequently, adding to the microbial load on kitchen surfaces (Flores et al. 2012). Composition of bacterial microbiota inside refrigerators appeared to be unique for each household, depending on the frequency of cleaning and types of stored foods (Jeon et al. 2013). Nevertheless, cold interiors of refrigerators and freezers were mostly dominated by Gram-positive, spore-forming representatives of *Firmicutes* (Flores et al. 2012), *Actinobacteria* and *Bacteroidetes* (Jeon et al. 2013). Genera *Pseudomonas*, *Pantoea*, *Sphingobacterium*, *Flavobacterium*, *Carnobacterium*, *Lactobacillus*, *Arthrobacter*, *Bacillus* and *Staphylococcus*, which dominated the interior of refrigerators (Table 1), were usually associated with stored food, especially untreated meat and vegetables (Jeon et al. 2013). There is a growing concern about food-borne pathogenic bacteria, such as *Listeria* spp., *Salmonella* spp., *E. coli* and *Pseudomonas* spp., which can be transmitted from food to refrigerator surfaces. Additionally problematic are too high temperatures inside refrigerators, which can promote growth of unwanted bacteria (Buchholz et al. 2011; Maktabi et al. 2013). Refrigerators and freezers are populated also by fungi. Currently, food and air appear to be the major source of fungi. Food-colonising fungi can pose a risk not only because of their potential virulence, but also due to their production of mycotoxins, some of which are detrimental for human health (NSF 2013). *Penicillium italicum*, *Botrytis cinerea*, *Mucor racemosus* and *Rhizopus oryzae* were isolated from the surfaces of refrigerators or from the air inside industrial freezers. Opportunistic pathogenic fungi belonging to the black yeasts *Cyphellophora*, *Exophiala*, *Knufia*, *Rhinocladiella* and *Ochroconis* were commonly isolated, in particular from rubber surfaces (Wang et al. 2018) and were additionally found, along with the genera *Acremonium*, *Alternaria*, *Aspergillus*, *Candida*, *Cladosporium* and *Scopulariopsis* on interior plastic parts and refrigerator ice and water dispensers (Table 1) (Fig. 2) (Altunatmaz et al. 2012).

### Washing machines

Laundry appliances, especially washing machines, are among the most common appliances used in households. Microbial presence inside washing machines was investigated for decades, mainly because of the risk of cross-transmission of pathogens via laundry. Gram-negative bacteria in planktonic form can survive washing temperatures of up to 50 °C, while Gram-positive bacteria can survive even higher temperatures, up to 60 °C (Munk et al. 2001). Thus, washing at low temperatures only reduces the number of live bacteria, both in water and on dirty clothes. Several studies reported colonisation of washing machines with bacteria from the genera *Acinetobacter*, *Bacillus*, *Clostridium*, *Corynebacterium*, *Escherichia*, *Micrococcus*, *Pseudomonas* and *Staphylococcus* (Table 1) (Perry et al. 2001; Panagea et al. 2005; Novak Babič et al. 2015). Their diversity varied depending on the water type and skin microbiota of the users (Callewaert et al. 2015). Particularly problematic is the presence of species *Clostridium difficile*, *Staphylococcus aureus*, *Klebsiella pneumoniae* and *P. aeruginosa* on laundry, since cross-contamination from laundry can occur, resulting in nosocomial infections (Rozman et al. 2013). Recently, a case report described a colonisation of newborns with the same genotype of *Klebsiella oxytoca* as was isolated from the detergent drawer and the rubber door seal of domestic washing machines (Schmithausen et al. 2019). Transfer and enrichment of some bacterial groups was shown also for cotton fabrics supporting the growth of the genera *Enhydrobacter*, *Corynebacterium* and *Acinetobacter*, while polyester fabrics in general favoured the growth of *Micrococcus* and *Staphylococcus* (Callewaert et al. 2015). Biofilm-forming genera such as *Pseudomonas* and *Shingomonas* frequently colonise plastic parts of the washing machine but are rarely transferred onto the laundry. However, their presence in washing machines causes persistent unpleasant odour due to bacterial degradation of detergents and other organic matter present on clothes and in the water and they are released into waste greywater, which exits the washing machines (Munk et al. 2001; Stapleton et al. 2013; Callewaert et al. 2015).

Although degradation of detergents, especially softeners, was reported also for various fungi, they are not involved in the development of unpleasant odour of clothes or washing machines (Stapleton et al. 2013; Novak Babič et al. 2015). Most fungi isolated from washing machines entered via tap water. They belong to the genera *Alternaria*, *Aspergillus*, *Candida*, *Capronia*, *Cladosporium*, *Cryptococcus*, *Fusarium*, *Naganishia*, *Penicillium*, *Rhodotorula* and *Trichosporon* (Table 1) (Fig. 2)*.* The most contaminated sites were plastic parts (e.g. drawers for detergents) and rubber seals (Gattlen et al. 2010; Kubota et al. 2012; Stapleton et al. 2013; Novak Babič et al. 2015). Recently, it was confirmed that drawers for detergent can be populated by biofilm-forming species, such as *Fusarium oxysporum* species complex, and emerging pathogen *C. parapsilosis*, as well as various black yeasts—*Exophiala*, *Knufia* and *Ochroconis* (Novak Babič et al. 2015, 2016a; Wang et al. 2018). However, the main concern in washing machines are the dermatophytic fungi and yeasts from the genera *Microsporum*, *Trichophyton* and *Candida*, due to their high infectivity rate and frequent cross-contamination of laundry (Tanaka et al. 2006).

### Clothes dryers

There are few reports on microbial contaminations of clothes dryers (tumble dryers). So far, it has been reported that in spite of high temperatures (between 50 and 70 °C) and consequently significant reduction in the number of viable microorganisms on fabrics during drying, microbial cross-contamination of laundry can occur. Bacteria, such as *Streptococcus pyogenes*, *Staphylococcus aureus* and *Bacillus* spp., can survive the high temperatures in dryers, resulting in clinical outbreaks especially in hospital environments (Brunton 1995; Fijan and Šostar Turk 2012). At the same time, we are not aware of any published study investigating the microbial load of the clothes dryer interior. Frequent consumer reports of the presence of an unpleasant odour or even visible dark biofilms inside dryers suggest potential fungal contaminations.

### Man-made materials as a new factor in selection of indoor water-borne microorganisms

Besides water availability, microbial colonisation is determined by the nutritional substances that can be derived from the building materials or environment (Gorny 2004), the presence of specific chemicals, such as surfactants from soap and shampoo (Hamada and Abe 2010) or from co-occurring microbial species. Understanding of the complex influence of materials on indoor microbiota will help us build indoor spaces and equip them in a way that will have predictable impacts on the diversity and abundance of microbes with the aim to minimise health hazards and promote human well-being (National Academies of Sciences).

In the role of different materials in promoting or preventing microbial disease, many studies have focused on objects acting as fomites, carriers of infectious agents and their possible antimicrobial properties. The longest reported periods of microbial persistence on surfaces are several months (Kramer et al. 2006) and while this is important in possible cross-contamination, the danger represented by the contaminated surface decreases with time. A quite different problem is microorganisms that actively grow indoors. The hot spots of these microorganisms are not so much the surfaces touched and contaminated by humans or animals, but much more the habitats with high availability of liquid water. The selection of materials in these habitats impeding or supporting microbial growth has exploded in the last decades—with some unpredicted consequences (Gostinčar et al. 2011). We now know that some biofilm-forming species including those pathogenic to humans can grow on polyethylene, polypropylene, rubber, stainless steel and other materials (Tsiaprazi-Stamou et al. 2019).

Building materials have always played a role in shaping indoor microbial communities. Even seemingly durable and inert materials such as stone are now known to support the growth of fungi, which can grow on them, form biofilms, penetrate into their structure and in the long term importantly contribute to their deterioration (Warscheid and Braams 2000). Fungal growth is possible on limestone and also on plaster, and several species have been shown to dissolve the CaCO_3_, among them strains of *Penicillium chrysogenum* and *Aspergillus versicolor* (Ponizovskaya et al. 2019). Newer, green building materials also present opportunities for growth of fungi, and as with conventional materials, with an external source of nutrients being an important factor contributing to growth (Mensah-Attipoe et al. 2015).

In the past, microorganisms in indoor environments could mostly choose between inorganic substrates such as stone or metal and much more easily degradable organic materials, often of plant origin. In the last few decades, however, another substrate has appeared and slowly started to dominate indoor spaces: synthetic polymers. These are also used in products designed to be in prolonged contact with water—water pipes, rubber seals, faucet aerators and many others. Produced in tens of millions of tons per year, different groups of polymers share one common trait: they are all highly resistant to biodegradation (Danso et al. 2019; SAPEA 2019). But just as some microbes can grow on glass, metals or silicon, others can grow on a range of organic surfaces such as plastic materials and other polymers (Isola et al. 2013). Additionally, plastic materials are completely inert. Some degraders of specific plastic polymers have already been found, particularly for polyurethane and polyethylene terephthalate (Danso et al. 2019; Narancic and O’Connor 2019), and while this degradation is in most cases slow and only partial, it may be enough to support slow growth of selected microorganisms. Furthermore, many of the frequently used plastics contain other chemicals in addition to the main polymer, which (among having other functions) act as fillers, stabilisers and plasticisers. These additives are often much smaller molecules, more amenable to microbial degradation (Danso et al. 2019). These substances can be seen as a new opportunity for the most adaptable and nutritionally versatile microbes, such as those exemplified by species that can use complex phenolic hydrocarbons as the sole source of carbon and energy (Prenafeta-Boldu et al. 2006).

With time, plastic deteriorates, with the resulting cracks and uneven surface providing a much larger surface area, which is also more difficult to clean. In nature as well as indoors, such plastics provide ample opportunities for microbial colonisation and this colonisation can contribute to further (bio) deterioration through physical, chemical and enzymatic means (Lucas et al. 2008). If a microorganism is to use the polymer (and not additives or external compounds) as a nutrient source, it then has to both depolymerise the large polymer molecules and also assimilate them (Shah et al. 2008). But even when microbes do not degrade plastics, plastics provide a very different surface for attachment than other materials. Bacterial biofilms on stainless steel were reported to be thinner and easier to remove than those found on polyethylene, which contained a higher proportion of viable bacteria—an important consideration when for example choosing the material of a kitchen sink, but also of tap water piping systems, where the formation of biofilms is linked with numerous problems impacting the safety of tap water. The difference was explained by differences in roughness, chemical composition and surface energy between both materials (Tsiaprazi-Stamou et al. 2019).

While microbial growth on plastics is generally seen as an aesthetic problem, it might have medical implications, too. Since the ability to degrade complex hydrocarbons, like stress tolerance, has been linked to fungal opportunistic pathogenicity, the fungi growing on plastic substrates may be selected for potentially more problematic species (Blasi et al. 2016; Gostinčar et al. 2018). Furthermore, in some circumstances, it can also be detrimental to the functionality of the plastic objects themselves. For example, some fungi known for their colonisation of synthetic polymers and production of aggressive metabolites are considered potentially problematic biocorrosive agents on the International Space Station and have caused problems on the orbital station Mir (reviewed in Novikova et al. 2006).

Another widely used material, providing a better surface for microbial attachment, is rubber, which is widely used to seal contacts between components of systems and appliances that need to be water-tight. In the investigation of materials used in tap water piping systems, rubber turned out to be by far the most problematic material in terms of biofilm formation (Szczotko et al. 2016). This matches well with the abundant fungal and bacterial colonisation of the rubber seals of dishwashers (Zalar et al. 2011; Zupančič et al. 2019).

Besides synthetic polymers, there are other compounds that have begun to be encountered by microbes indoors in the last decades and which have not been generally available in the past. Synthetic detergents used in soaps and shampoos are used widely and besides other sources of organic compounds (such as food in the kitchen, desquamated skin cells in the bathroom) provide a regular source of nutrients. Many of them are easily biodegradable and this is so by design. After the visible foaming pollution in streams and wastewater treatment plants in the 1940s and 1950s, more degradable compounds were produced and in many regions the use of biodegradable detergents is now obligatory by law. Many surfactants degrade even before reaching the wastewater treatment plant (Cowan-Ellsberry et al. 2014; Merrettig-Bruns and Jelen 2009). Their remnants can be used by microorganisms as nutrient sources. Several of the fungi that are often isolated from bathrooms, *Cladophialophora boppii*, *Exophiala spinifera*, *Exophiala salmonis*, *Phialophora europaea*, *Phoma herbarum* and *Scolecobasidium constrictum*, are able to grow on a nonionic surfactant (polyoxyethylene (Danso et al. 2019) lauryl ether) used in shampoos as a nutrient source, while *Aureobasidium* sp. and *Cladosporium cladosporioides* can use sodium salts of fatty acids (Hamada and Abe 2010).

Commercial products containing detergents often also contain preservatives that hamper microbial growth (Smaoui and Hlima 2012). Infections connected with the use of non-medical liquid soaps in the medical settings have been ascribed to the packaging and dosage system rather than the soap itself, since in the soap any microbial contamination diminishes with time, for bacteria quicker than for fungi (Tyski et al. 2013). However, dilution of the soap, either by the user or by the producer, will also impair its antimicrobial properties and such soap has been shown to support the survival of bacteria such as *K. oxytoca*, *Serratia liquefaciens*, *Shigella sonnei*, *Enterobacter cloacae*, *E. gergoviae* and *Serratia odorifera* (Schaffner et al. 2018). Microorganisms can also grow in the soap water with which we wash them from our skin or objects. *Escherichia coli* and *Enterococcus faecalis* were shown to grow in the rinses of various soaps, except those containing triclosan, and chlorhexidine gluconate, respectively (Pérez-Garza et al. 2017).

### Clinical relevance of polyextremotolerant fungi found indoors

Infection of mammal hosts is a rare ability in the fungal kingdom. Of the 140 orders distinguished based on current taxonomy, only 15% contain species that repeatedly show this infectious potential (Gostinčar et al. 2018). Fungi that successfully infect a human body have to overcome several obstacles, such as high temperature (Robert and Casadevall 2009), oxidative bursts of human phagocytes and severe iron limitation (Hamad 2008; Kumamoto 2008), and low water activity and low pH in case of skin penetration (Elias 2007). Only a handful of true fungal pathogens evolved virulence factors enabling them to infect healthy individuals. Although the list of opportunistic fungi is much longer, they cause infections only sporadically, primarily in immunocompromised hosts. It has been shown that traits promoting virulence in opportunists have likely evolved for enabling the survival of the fungus in the environment (Song et al. 2017), primarily in stressful conditions (Gostinčar et al. 2011). For example, in the known pathogen *Cryptococcus neoformans*, the mechanisms that enable its survival during infection probably evolved in response to stress in its primary ecological niche, bird manure (Brown et al. 2007). Adaptations to stress encountered outside the mammalian host (exaptations—i.e., mechanisms that originally evolved for different purposes) can unintentionally facilitate the fungal establishment in the host (van Burik and Magee 2001; Casadevall 2007).

Although not the topic of this paragraph, some parallels can be found with the behaviour of bacterial opportunists, which has in many cases been studied for much longer than fungi. Perhaps one of the most obvious examples is *Pseudomonas aeruginosa*, a bacterium also tightly connected to habitats with good accessibility of water, such as faucets and drains. Similarly to fungal opportunists, it can tolerate various stresses (although not desiccation) and forms biofilms and causes a wide spectrum of diseases in immunocompromised patients (Bédard et al. 2016).

One of the most remarkable demonstrations of fungal stress tolerance is the growth of some fungi in extreme environments, in which by definition most microbial species cannot survive. Two modes of adaptation to extreme conditions were recognised. On the one hand, specialised extremophilic and extremotolerant species evolved to efficiently cope with one major specific stress factor and are thus limited in their capacity for habitat shifts. Polyextremotolerant species on the other hand tolerate many different types of stress and are often extremely adaptable (Gostinčar et al. 2010), prone to habitat shifts and colonisation of novel, including man-made indoor habitats, as exemplified by black yeasts *A. melanogenum* and *E. dermatitidis* (Gostinčar et al. 2011; Zalar et al. 2011; Hamada and Abe 2010; Lian and de Hoog 2010). Based on the assumption that exaptations might be the link between polyextremotolerance and opportunistic pathogenicity, a kingdom-wide phylogenetic analysis showed a statistically significant co-occurrence of extremotolerance and opportunism at the level of fungal orders (Gostinčar et al. 2018).

The main traits that define fungal opportunism and colonisation of stressful indoor environments are thermotolerance in combination with osmotolerance/osmophily, and oligotrophism, together with siderophore production at 37 °C, production of different hydrolytic enzymes, urease activity, use of different hydrocarbons as sole carbon source, tolerance to oxidative stress, melanisation and biofilm production (de Hoog et al. 2005; Gostinčar et al. 2018; Lavrin et al. 2020). While some of these traits are known for *Candida* spp., *Fusarium oxysporum* (FOSC) and *F. solani* complexes (FSSC), all of these are characteristic for the black yeast *E. dermatitidis*, dominant fungus in dishwashers around the world (Zalar et al. 2011; Lavrin et al. 2020), and *A. melanogenum* prevailing in tap water (Novak Babič et al. 2016b, 2017a).

Tolerance to oxidative stress is tightly linked to the lifestyle of polyextremotolerant fungi as shown for different polyextremotolerant yeasts, which contain numerous homologues of the three major enzymes involved in cellular oxidative stress responses (i.e. catalases, SODs, peroxiredoxins) (Gostinčar and Gunde-Cimerman 2018). Oxidative stress can be triggered both by abiotic stressors (high salinity, extreme temperatures, starvation, light, mechanical damage and other) (Gostinčar and Gunde-Cimerman 2018) and during infection. Oxidative burst is a crucial part of animal and plant immune responses (Hamad 2008; Kumamoto 2008), and fungal ability to tolerate oxidative bursts protects them against attacks by neutrophils (Leal et al. 2012).

Many polyextremotolerant fungi are additionally protected by melanisation of the cell walls. Melanin shields fungal cells by acting as a scavenger of reactive oxygen species, protecting them against different types of abiotic stress (Slepecky and Starmer 2009; Gostinčar et al. 2012; Kogej et al. 2007; Kejžar et al. 2013) and increases their resistance to lysis, phagocytosis and to clinically used antifungal agents (van Baarlen et al. 2007; Feng et al. 2001; Nosanchuk et al. 2015; Schnitzler et al. 1999; Lavrin et al. 2020).

Polyextremotolerant species use various mechanisms to overcome starvation, both in nature and in oligotrophic indoor water-related environments. This exaptation is advantageous also in the mammalian host, where nutrient limitation is an important defence mechanism. This is clearly demonstrated in the case of iron. During an infection in mammals, phagocytes restrict fungal growth by releasing mediators that sequester iron (Kumamoto 2008), while in extreme environments its availability is limited due to competing microbes and often by the generally low nutrient concentrations as well. Only fungi that can produce high-affinity iron-chelating compounds, such as siderophores, as exemplified by *A. pullulans*, can thrive in iron-limited conditions (Neilands 1993; Johnson 2008; Zajc et al. 2019).

A crucial step in the colonisation of any kind of surface is the adhesion of the microbial cells to the surface and formation of a biofilm—a consortium of microorganisms embedded in extracellular polymeric substances. Mature biofilms provide increased resilience to any attack by the host immune defences, to antimicrobial agents (Fanning and Mitchell 2012; Inci et al. 2012) and to different abiotic stressors present in extreme environments (Zajc et al. 2019; Zupančič et al. 2018).

Survival in animal hosts and extreme environments is also linked to the production of a wide repertoire of extracellular metabolites, i.e. the secretome. Urease activity is for example directly linked to animal pathogenesis in bacteria and fungi (Cox et al. 2000; Rutherford 2014). Increased pH due to urease activity reduces acidification and maturation of phagolysosomes, thus impairing pathogen killing and antigen presentation (Cox et al. 2000; Rutherford 2014). Proteases and lipases are well-known virulence factors that facilitate the spread of the pathogen through tissues or neutralise the components of the immune system (Singaravelu et al. 2014).

Some polyextremotolerant fungi are able to assimilate monoaromatic hydrocarbons as carbon sources, and these species are frequently encountered both in outdoor hydrocarbon-polluted environments and indoors in plastic-rich niches (Döğen et al. 2013a, b; Seyedmousavi et al. 2011; Zhao et al. 2010; Isola et al. 2013; Chandran and Das 2012). A physiological connection between hydrocarbon assimilation and neural infection was suggested for certain polyextremotolerant black yeasts (Prenafeta-Boldu et al. 2006; Lavrin et al. 2020).

While the above described traits are shared among many polyextremotolerant fungi, it appears that the decisive fungal virulence factor is thermotolerance, i.e. the ability to thrive at 37 °C and above (Robert and Casadevall 2009; Chandran and Das 2012; Gostinčar et al. 2018). This generally rare ability among fungi might become more frequent in the future, due to increased urbanisation and increased occurrence of high-temperature domestic environments and thermal adaptations of fungi on a global scale (Gostinčar et al. 2011; Lavrin et al. 2020).

Thermotolerance being the crucial virulence factor is in line with the observation that opportunists and non-opportunists cannot be distinguished on the level of genomic signatures associated with pathogenicity (Gostinčar et al. 2018). The number of genes for known virulence factors, such as enzymes for secondary metabolite production, carbohydrate-active enzymes (CAZymes), small secreted proteins, peptidases and proteases (Monod et al. 2002; Fischbach and Walsh 2006; van Baarlen et al. 2007; Feng et al. 2001; Nosanchuk et al. 2015; Schnitzler et al. 1999), nonribosomal peptide synthetases (NRPSs) involved in the synthesis of siderophores (Bushley and Turgeon 2010; Silva et al. 2011) and toxins (Walton 1996; Haese et al. 1993), all thought to be important for virulence and resistance to clinically used antifungal agents, was the same in opportunistic and other species (Langfelder et al. 2003; Schmaler-Ripcke et al. 2009). However, polyextremotolerant and opportunistic pathogenic fungi repeatedly share a common phylogenetic history, supporting the hypothesis that traits important for fungal pathogenicity are shaped also by selection pressures outside of the host, acquiring “accidental virulence” (Casadevall and Pirofski 2007). The overview of indoor opportunistic polyextremotolerant fungi showed that their distribution in the fungal tree of life is not even, corresponding to the repeated emergence of opportunistic pathogens in some groups, but not in others.

Many species of black fungi are now increasingly being recognised as medical problems (Silveira and Nucci 2001; Chowdhary et al. 2014; Lavrin et al. 2020). Greater numbers of susceptible hosts, improved diagnostics and recognition of water-related indoor microbiomes and changes of our lifestyle have all been proposed as reasons for this trend (Casadevall et al. 2011; Gostinčar et al. 2011, 2015). It appears that these fungi do not specialise for pathogenicity as such. Instead, the evolution of their invasive potential is uncoupled from their hosts and tightly linked to their polyextremotolerant ecology, the same ecology that is also behind their ability to inhabit and thrive in various indoor habitats and use the opportunities created by the changes in human lifestyle and indoor environments.

## Conclusions

Good personal hygiene practices, proper hygiene of household surfaces and regular maintenance of household appliances can substantially lower the presence of indoor water-related microorganisms and can prevent most infections (Flores et al. 2012). Some microorganisms, however, are capable of surviving even rigorous sanitation treatments (Cooper 2010). This should be taken into consideration especially in households inhabited by immune-compromised people (Novak Babič et al. 2017a, b).

Microbiological analyses of water quality are typically limited to certain standard procedures and regulations, which are often outdated even before they are even generally accepted by international and national regulating bodies. Standard microbiological tests are—with few exceptions—designed to control the presence of the most medically relevant bacteria that represent health risks to humans and animals. However, a much less controlled problem is the emergence of new bacterial and fungal pathogens. These can be present in disinfected water, in long-lived biofilms inside the plumbing systems, inside household appliances and on other surfaces, particularly in kitchens and bathrooms. Here, microorganisms do not just persist, but may spread further within the household, survive wastewater treatments and return to the environment, potentially creating a microbial vicious circle.

Microorganisms are introduced into domestic appliances via water, air, dishware, food, hands and clothes. Of particular concern are polyextremotolerant microbes that can adapt to conditions inside household appliances (that are now also becoming common in less developed countries (ConsumerReports 2016; SURS 2020). Although appliances were designed to help consumers maintain cleanliness, biofilm formation inside their interiors cannot be prevented. These biofilms can be established on interior materials due to the constant presence of water, either entering the appliance from the tap water distribution system or condensing on the surface. Roughness of the materials, hydrophobicity and resistance of materials to microbial attack play important roles in biofilm establishment. Biofilms contain high concentrations of microbial cells, which can be sporadically released as aerosols into the air or as individual planktonic cells or clumps of cells into the water. These microbes could represent a hitherto largely overlooked danger for infection through inhalation of contaminated aerosols, trauma or contact with infected surfaces (Novak Babič et al. 2017a, b).

Two modes of adaptation can be recognised in microorganisms inhabiting humid indoor habitats (water, wet cells and household appliances). One is specialisation to a specific stress factor, a so-called monoextremotolerance, which is typically linked to limited capacity for habitat shifts. Such microorganisms are primarily found in refrigerators. Polyextremotolerant fungal species on the other hand tolerate many different types of stress and are often extremely adaptable and can populate household appliances such as dishwashers and washing machines in large numbers (Gostinčar et al. 2010). Such large adaptability and stress-tolerance is thought to be linked to the great capacity of these fungi to colonise novel and generally inhospitable habitats, including the human body (Gostinčar et al. 2011). It is thought that adaptations to stress encountered outside the mammalian host evolved to promote the survival of the microbes in the environment, but can also serve as exaptations and thus (as a side effect) occasionally allow the establishment of some fungi in the host (van Burik and Magee 2001; Casadevall 2007). Many species of black extremophilic fungi are now being recognised as a medical issue (Silveira and Nucci 2001; Chowdhary et al. 2014). These black yeasts did not specialise for pathogenicity as such, but their invasive potential is tightly linked to their polyextremotolerant ecology and most likely uncoupled from their host-microbe relationship.

The present studies show that water is an important source of exposure to environmental microorganisms in domestic environments and that the selection of water-borne microorganisms during water treatment and upon entering the generally inhospitable indoor environment favours species with a higher virulence potential. While the number of studies supporting this observation is steadily increasing, more epidemiological and experimental approaches are needed to further elucidate the role of the domestic water microbiome in human health. With an ever-growing fraction of the world population living in megacities and spending a majority of their lives indoors, understanding the role of the complex household microbiome—beyond the few primary pathogens—is becoming increasingly urgent.
